# Thermal BCS-BEC Crossovers in Finite Systems

**DOI:** 10.3390/e27111116

**Published:** 2025-10-30

**Authors:** Angelo Plastino, Flavia Pennini, Victor Apel

**Affiliations:** 1 Instituto de Física La Plata–CCT-CONICET, Universidad Nacional de La Plata, C.C. 727, La Plata 1900, Argentina; angeloplastino@gmail.com; 2 Departamento de Física, Universidad Católica del Norte, Av. Angamos 0610, Antofagasta 1270709, Chile; vapel@ucn.cl; 3 Departamento de Física, Facultad de Ingenieria, Universidad Nacional de Mar del Plata (UNMDP), CONICET, Av. J.B. Justo 4302, Mar del Plata CP 7600, Argentina

**Keywords:** BCS-BEC crossover, fermionic pairing correlations, thermodynamic response functions, finite-size systems, *SU*(2) × *SU*(2) model, thermal fluctuations, statistical quantifiers, mesoscopic quantum systems

## Abstract

We investigate the thermal evolution of fermionic pairings in a finite-size *SU*(2) × *SU*(2) complex model, drawing an analogy to the BCS-BEC crossover in interacting quantum gases. Unlike the conventional crossover, which is driven by tuning the interaction strength, our study suggests that temperature alone can induce a smooth transition from weakly bound Cooper pairs (BCS-like state) to tightly bound dimers (BEC-like state). Using an exactly solvable model with a finite number of fermions, we analyze the structure of eigenstates, pairing correlations, and thermodynamic response functions. We demonstrate that different multiplet structures, characterized by distinct quasi-spin quantum numbers, become thermally accessible, effectively mimicking the crossover behavior seen in ultracold Fermi gases. Our results provide new insights into the role of thermal fluctuations in quantum pairing phenomena and suggest alternative routes for exploring crossover physics in mesoscopic and strongly correlated systems.

## 1. Introduction

The BCS-BCE transition refers to the phenomenon where a system transitions from the Bardeen–Cooper–Schrieffer (BCS) state, characterized by weakly correlated pairs of fermions (Cooper pair), to the Bose–Einstein condensation (BEC) state, where fermionic pairs condense into a single quantum state. This transition may also be achieved by tuning the interaction strength between fermions, which can be carried out through several methods. The BCS-BCE crossover is a smooth transition rather than a phase transition, meaning that there is no abrupt change in the order of the physical system when transitioning from one phase to the other. This transition is also significant in understanding the behavior of strongly correlated systems and has implications for various applications in condensed matter physics and quantum technologies.

Thus, one describes the smooth evolution between two distinct regimes of fermionic pairing: the transition from the weakly bound Cooper pairs in the BCS regime to the strongly bound dimers forming a BEC state. This concept has gained significant attention in condensed matter and ultracold atomic physics, as it provides a unified framework for understanding superconductivity, superfluidity, and strongly correlated quantum gases [1,2,3,4,5,6,7,8,9,10,11,12].

Recall that in quantum physics, the coherence length is the distance over which a quantum system, such as a wave function, maintains a stable and well-defined phase relationship. It measures how far a quantum entity can propagate while still exhibiting clear, predictable wave-like properties, allowing for stable interference effects. A longer coherence length indicates a more stable, sinusoidal wave and a higher degree of quantum coherence, which is crucial for quantum phenomena like entanglement and interference.

In the BCS limit, attractive interactions between fermions lead to the formation of extended, overlapping Cooper pairs. These pairs are highly delocalized, and their condensation results in superconductivity or superfluidity. The pairing mechanism is driven by a weak attractive interaction that is mediated, for example, by phonons in conventional superconductors. In this regime, the coherence length of the pairs is much larger than the interparticle distance, and the many-body wavefunction is well described by BCS theory [1,2,3,4,5,6,7,8,9,10,11,12].

On the other hand, in the BEC limit, the attractive interaction is strong enough to bind fermions into tightly bound molecules, which behave as bosons. These bosonic molecules undergo Bose–Einstein condensation at low temperatures, forming a superfluid state similar to that observed in dilute Bose gases. In this regime, the binding energy of the pairs is large, and the coherence length becomes much smaller than the interparticle spacing. When the coherence length in a BEC state becomes much smaller than the interparticle spacing, this signifies the system is transitioning from a coherent, collective state (like a true BEC) to a state where particles are more localized and individually distinguishable, despite being bosons. This can happen at higher temperatures or with strong particle interactions, leading to increased phase fluctuations and a loss of macroscopic quantum coherence, effectively behaving more like a collection of individual atoms rather than a single quantum wave.

In this work, we propose an alternative CBS-BCE route where temperature itself acts as the tuning parameter for the crossover. Although temperature-driven crossovers are well known in classical fluids, such as in the supercritical liquid–gas region where the Widom line separates liquid-like from gas-like behavior [13,14,15], our approach demonstrates an analogous phenomenon in a finite quantum SU(2) fermionic model. Here, thermal fluctuations reorganize the microstate populations, yielding a smooth, collective transition reminiscent of classical critical crossovers that is rooted in quantum pairing dynamics.

Moreover, modern research has revealed that noise and fluctuations can play constructive roles in quantum systems. Phenomena such as noise-enhanced stability (NES) [16,17,18] and correlated quantum noise [19] demonstrate that stochastic effects may extend coherence lifetimes and even stabilize ordered phases. This broader perspective, emphasized in Parisi’s Nobel Lecture [20], highlights the universality of fluctuation-driven organization across classical and quantum domains. By situating the present model within this context, we underscore temperature-induced coherence as part of a larger class of fluctuation-enabled transitions.

Noise-enhanced sensitivity refers to the counterintuitive phenomenon whereby a finite amount of noise increases a system’s responsiveness to a control parameter. In the present SU(2) mode, NES can appear because moderate thermal or external noise activates transitions between nearly degenerate many-body states.

To describe the system quantitatively, we adopt an exactly solvable Lipkin-type Hamiltonian expressed in SU(2)×SU(2) form. The canonical ensemble is employed throughout, since the total particle number *N* is fixed; temperature only redistributes probabilities among paired and unpaired configurations within the *N*-conserving manifold. This guarantees thermodynamic consistency while allowing the model to emulate pairing dynamics similar to grand canonical BCS treatments.

### 1.1. Relation to BEC-BCS and Excitonic Insulator Literature

It is important to situate our findings within the long tradition of studies on fermionic pairing and condensation. Early theoretical work on excitonic condensation (often termed the “excitonic insulator”) by Keldysh and Kopaev [21] and the complementary analyses of Jérôme, Rice, and Kohn [22] established how attractive interactions between fermionic species can drive population-scale instabilities and ordered phases. Later developments formalized the smooth crossover between weak-coupling BCS pairings and the strong-coupling Bose–Einstein condensation (BEC) of tightly bound pairs; a paradigmatic treatment of this crossover at finite temperatures is given in the influential work of Nozières and Schmitt-Rink [23] and in subsequent reviews (see, e.g., [24,25]).

While these studies address density- and temperature-driven pairing phenomena in extended systems and provide key insights into macroscopic condensation, our contribution differs in crucial respects. First, we consider finite, few-fermion ensembles (two energy levels, spin–flip interaction) and focus on information-theoretic observables (entropy and statistical complexity). Second, the control parameter in our work is the microscopic interaction’s strength *G* (at a fixed inverse temperature β) rather than particle density or chemical potential. We illuminate the finite-size effects tied to level rearrangements and the discrete spectrum of the model. Accordingly, rather than reproducing the BEC–BCS crossover physics in the thermodynamic limit, our present results will offer a complementary, finite-*N* perspective: they will reveal how many-body sensitivity and informational content are reorganized as the interaction is tuned.

### 1.2. Our Aim and Motivation

In this work, we propose an alternative route to explore the BCS-BEC crossover, not by varying interaction strength but by considering its temperature dependence in a finite-size *SU*(2) × *SU*(2) model [12]. The model naturally incorporates pairing correlations and allows us to study how thermal effects lead to a gradual transition from weak to strong pairings as temperature increases. Our results suggest that temperature itself can act as a tuning parameter for the crossover in mesoscopic systems, revealing new insights into the role of finite-size effects in quantum pairing phenomena. The smooth evolution between the Bardeen–Cooper–Schrieffer (BCS) regime of extended, overlapping Cooper pairs and the BEC state regime of tightly bound fermion pairs represents one of the most profound unifying concepts in modern many-body physics [8]. It provides a common framework for understanding phenomena as diverse as ultracold Fermi gases, high-$T_{c}$ superconductors, and even aspects of nuclear matter and neutron stars. However, the BCS-BEC crossover is most often studied through approximate mean-field approaches or numerical simulations, where disentangling finite-size effects, interaction-driven mechanisms, and thermal fluctuations can be challenging.

In this context, exactly solvable pairing models based on SU(2) algebras offer a uniquely transparent laboratory. They allow us to examine how fermionic pairing evolves with system size and temperature and separate the structural effects of the Hilbert space from the dynamical ones induced by interactions. The *SU*(2) × *SU*(2) construction [12], in particular, provides closed-form eigenstates and thermodynamic quantities, ensuring that the signatures we identify are not artifacts of approximation schemes. This clarity is especially valuable when addressing mesoscopic systems, where the interplay of thermal fluctuations and pairing correlations may mimic or even induce crossover-like behavior.

Studying the BCS-BEC transition in such an exactly solvable framework [12] is thus important for two reasons. First, it yields benchmark results that can guide the interpretation of experiments and simulations in more complex systems. Second, it offers new insights into unconventional routes to crossover physics, such as the possibility—highlighted in this work—that temperature alone may drive a transition from BCS-like to BEC-like behavior even in the absence of tunable interactions. By grounding the analysis in an exactly solvable model, we expose the universal structural mechanisms of pairing crossovers, helping to connect nuclear, condensed matter, and cold-atom physics within a common theoretical language.

## 2. Present Scenario

As we saw above, crossover superconductivity, specifically the BCS-BEC crossover, refers to the transition between two different theoretical models that describe superconductivity. In conventional superconductors, the BCS (Bardeen–Cooper–Schrieffer) theory explains superconductivity as arising from weakly interacting fermion pairs (Cooper pairs). As the interaction between fermions increases, the system can transition towards a BEC (Bose–Einstein condensation) regime where tightly bound fermion pairs behave more like bosons and condense into a superconducting state. This crossover between the BCS and BEC descriptions is crucial for understanding superconductivity in various materials, including some high-temperature superconductors. We will study the crossover here in the framework of an exactly solvable Lipkin model [26].

### 2.1. Our Exactly Solvable Model

Bohr, Mottelson, and Pines [4], as well as Belyaev [5], were able to successfully adapt to the superconductivity techniques advanced by Bardeen, Cooper, and Schrieffer [6,7]. Nuclear superconductivity on the basis of nuclear fermion-pairing is common fare in nuclear structure descriptions [8], where one works with finite systems [8].

In this work, we unveil peculiar temperature and number of fermion effects displayed by *N*−fermion, which is described by an exactly solvable Lipkin model. Among hundreds of papers on nuclear superconductivity see, in addition to [8], see for example, Refs. [9,10,11].

The effects we wish to report involve a context delineated by the Gibbs canonical ensemble, which is focused on an exactly solvable model introduced in Ref. [12], for which the BCS treatment of superconductivity becomes exact; this reporting choice is was made so that our results are not based on approximate treatments. We start by presenting such a model below.

### 2.2. The Mother Model: That of Lipkin et al. (LM) [26]

This LM [26,27] was exceedingly helpful in a multitude of investigations regarding the validity and adequacy of different many-body techniques devised to inspect and explore the manifold traits of the quantum many-body problem.

The LM template is constructed as an SU(2) algebra associated with particular operators denominated as quasi-spin ones and can produce easily attainable exact solutions to the relevant Schröedinger equation (SE). These exact solutions are to be compared with the approximate ones encountered using diverse kinds of theoretical approximations to the problem at hand. In the LM context, one speaks an “angular momentum” language. The relevant Casimir operator is akin to $J^{2}$ [26] and is attached to it several multiplets. Lipkin et al. only used the multiplet associated to the system’s ground state [26]. For facing superconductivity, however, one needs additional multiplets. A practical way of handling them was advanced in reference [12].

As stated above, Cambiaggio and Plastino (CP) [12] proposed a simple LM-extension that allowed for the formulation, in quasi-spin parlance, of a BCS-like construct mimicking ordinary superconductivity. The construct yields exact solutions. This extension is a generalization of the SU(2)’s LM to an SU(2)×SU(2)-related alternative, involving a variable fermion number.

### 2.3. Mathematics of the SU(2)×SU(2) Model for N Fermions

This construct [12,28] focuses on *N* fermions disseminated in a couple of (N=2Ω) (2Ω)-fold degenerate single-particle levels. The two levels are separated by an energy gap ε. One characterizes the 4Ω multiplicity via the quantum numbers *p* and μ in the following manner: p=1,…,2Ω, and μ=±1. One deals with the angular momentum SU(2) quasi-spin operators as follows: [26]

(1) $$J_{z}=\left(1/2\right)\sum_{p,\mu }\mu C_{p,\mu }^{+}C_{p,\mu },$$

(2) $$J_{+}=\sum_{p}C_{p,+}^{+}C_{p,-},$$

(3) $$J_{-}=\sum_{p}C_{p,-}^{+}C_{p,+}.$$

Cambiaggio and Plastino supplemented them with the SU(2) “pairing” operators:

(4) $$Q_{0}=\left(1/2\right)\sum_{p,\mu }C_{p,\mu }^{+}C_{p,\mu }-\Omega ,$$

(5) $$Q_{+}=\sum_{p}C_{p,+}^{+}C_{p,-}^{+},$$

(6) $$Q_{-}=\sum_{p}C_{p,-}C_{p,+}.$$

Evidently, $Q_{+}$ creates and $Q_{-}$ annihilates two fermions yielding zero contribution to the values of the operator $J_{z}$. We say then that these two fermions “couple” to $J_{z}=0$. Note that any *Q* operator will commute with all *J* operators and vice versa (SU(2)×SU(2)). We have a complete orthonormal basis determined by the eigenvalues of $J^{2},J_{z},Q^{2}$, and $Q_{0}$, i.e., $|J,Q,J_{z},Q_{0}\rangle$. A “pairing” Hamiltonian commuting with the number of fermions operator reads [8] (we take ε=1)

(7) $$H=\varepsilon J_{z}-\left(G/2\right)Q_{+}Q_{-},$$

with *G* denoting the pairing strength. Notably, in this paper, we sometimes take G=0. The pairing interaction above has exactly the same appearance as that used in nuclear theory [see [8], Equation (4.140)]. An important quantity for our purpose is the quasi-spin seniority number ν:

(8) ν=2(Ω−Q),

which is the number of particles not “paired” with $J_{z}=0$. In other words, ν yields the number of “unpaired” particles in a *Q* multiplet. As for our multiplet structure, one has [12]

(9) J=ν/2,

while

(10) J+Q=Ω.

In the case of the Lipkin model, we have N=2Ω and $Q_{0}=0$, which are equalities that are also used in this work [12].

At zero temperature, the unperturbed ground state (no interaction) has J=Ω, $J_{z}=-\Omega ,\;\;Q=Q_{0}=0$, belonging to the multiplet $J=\Omega ,\;\;Q=Q_{0}=0.$ We represent the exact eigenvalues of *H* as [12]

(11) $$E\left(J,Q,J_{z},Q_{0}\right)=J_{z}-\left(G/2\right)\left[Q\left(Q+1\right)-Q_{0}\left(Q_{0}-1\right)\right].$$

The energy of the unperturbed ground state ($\nu =N,Q=Q_{0})$ is [12]

(12) $$E_{0}=-\Omega .$$

It is important to note that the state of quasi-spin seniority zero, for which all fermions are “paired” with $J_{z}=0$, faithfully mimics a nuclear “superconducting” state [12,29,30], characterized by ν=0 and Q=Ω. Its superconducting energy reads

(13) $$E_{s}=-\left(G/2\right)\left[\Omega \left(\Omega +1\right)-Q_{0}\left(Q_{0}-1\right)\right],$$

(14) $$E_{s}=-\left(G/2\right)\left[\Omega \left(N-\nu \right)+\frac{\nu }{2}\left(\frac{\nu }{2}-1\right)-\frac{N}{2}\left(\frac{N}{2}-1\right)\right].$$

The pertinent state metamorphoses into the superconducting state of the pairing-interacting model when

(15) $$G=G_{\mathrm{crit}}\geq \left(4\Omega /N\right)\frac{1}{2\Omega +1-N/2},$$

Whenever $G<G_{\mathrm{crit}}$, the system remains in the unperturbed ground state (UGS). Note that the greater *N* is, the less “labor” is needed, measured in *G* units, to force the system to be a superconductor—a nice fact that was not duly emphasized in Ref. [12] but is critical for our present purposes. In other words, as *N* grows, $G_{\mathrm{crit}}$ diminishes.

### 2.4. The Role of Quantum Noise and Stochastic Fluctuations in the Pairing Strength

We introduce a weakly time-dependent stochastic correction to the coupling constant

$$G\to G+\delta G\left(t\right),\;\langle \delta G\left(t\right)\delta G\left(t^{\prime }\right)\rangle =\sigma_{G}^{2}\delta \left(t-t^{\prime }\right),$$

and analyze how such fluctuations modify the Fisher information and Binder cumulant behavior. This formulation clarifies how moderate coupling randomness smooths sharp crossover signatures while enhancing the persistence of pair correlations, a behavior analogous to noise-assisted ordering.

We also clarify Equation (15) and now provide the following explicit definition:

(16) $$G_{\mathrm{crit}}=\frac{\Delta E}{2N},$$

where ΔE is the single-particle level spacing between the two degenerate subspaces. For $G>G_{\mathrm{crit}}$, pairing correlations dominate, marking the onset of the BEC-like regime.

### 2.5. SU(2)×SU(2) Model and Finite Temperatures

At finite temperature *T*, with β=1/T, the remnants of the T=0-phase transition from the unperturbed state to superconductivity are now called crossovers [31]. For our SU(2)×SU(2) model, the pertinent Gibbs canonical ensemble treatment was devised and implemented in Refs. [29,30].

In investigating ground states, only the J+Q=Ω “band” needs consideration. For finite *T*, instead, all states belonging to different bands are now “accessible” in the pertinent Gibbs’ statistical ensemble. For writing the partition function *Z*, one needs the degeneracy Y(J,Q) given in Refs. [29,30]:

(17) $$Y\left(J,Q\right)=\frac{\left(2\Omega +2)!(2\Omega )!(2J+1)(2\Omega +1\right)}{(\Omega +J+Q+2)!(\Omega +J-Q+1)!(\Omega -J+Q+1)!(\Omega -J-Q)!}.$$

We begin with a partial partition function $Z_{M}$ of the type [29,30]

(18) $$Z_{M}=\sum_{M=-J}^{M=J}\;\exp\left[-\beta \left(M-\frac{G}{2}Q\left(Q+1\right)\right)\right],$$

and then, the true partition function *Z* becomes

(19) $$Z=\sum_{J,Q}\;Y\left(J,Q\right)Z_{M},$$

where *J* and *Q* run over all values that verify the following restrictions [27,29,30]:

(20) 0≤J≤Ω,

(21) 0≤Q≤Ω,

(22) 0≤J+Q≤Ω.

Now, we proceed to evaluate, from *Z*, our statistical quantifiers. We will first need to modify (22) to the form

(23) 0≤J+Q=s≤Ω.

When performing the double sum over *J* and *Q* for *Z*, it is convenient to sum over J+Q=s and *J*. Additionally, *Q* remains fixed at Q=s−J, where

(24) 0≤s≤Ω,

and s=0,1,2,3,…,Ω. Also, J=0,1,2,…,s. Accordingly,

(25) $$Z=\sum_{J,Q}\;Y\left(J,Q\right)Z_{M}=\sum_{s=0}^{\Omega }\sum_{J=0}^{s}\;Y\left(J,Q\right)Z_{M}.$$

We remind the reader that N=2Ω. Gibbs’ canonical probability distribution (CPD) reads

(26) $$P\left(M,Q,J\right)=\frac{Y\left(J,Q\right)\exp\left[-\beta \left(M-\frac{G}{2}Q\left(Q+1\right)\right)\right]}{Z}.$$

Note that for Q=0, there are no coupled pairs of fermions. This is the case of the ground state multiplet. All excited multiplets have paired fermions and, thus, partial superconductivity. These excited multiplets become available for occupation as soon as the temperature is different from zero.

### 2.6. Superconductivity Index

The superconductivity index X is a statistical measure of the fraction of fermions that are thermally organized into paired configurations. Now, denote $P^{†}$ as the creation operator for a pair of fermions and *P* as the associated destruction operator. Then, it is defined as

(27) $$X=\frac{\langle P^{†}P\rangle }{N/2},$$

where $P^{†}P$ counts the correlated fermion pairs. Hence, *X* measures the population imbalance between paired and unpaired fermions, and it is directly proportional to the average pair of coherence strengths.

In physical terms, *X* serves as a normalized indicator of the BCS-order parameter magnitude within the finite SU(2) manifold:

- For $T\ll T_{c}$, X≃1, corresponding to a nearly complete pairing (BCS-like regime);
- As *T* increases, *X* decreases smoothly, marking progressive pair breaking;
- For high *T*, X→0, corresponding to the unpaired (normal) state.

Explicitly [32], for the effective superconductivity index *X*, we write [Cf. Equation (8)]

(28) $$X=\sum_{J,Q}\sum_{M=-J}^{J}P\left(M,Q,J\right)\;\left(\frac{N-\nu }{N}\right),\;\nu =2\left(\Omega -Q\right),\;N=2\Omega ,$$

where P(J,Q,M) denotes the canonical probabilities of the Dicke-like multiplets (J,Q,M). It is equal to unity for a perfect superconductor and vanishes for the unperturbed system. As a consequence,

(29) $$\langle \nu \rangle =1-X=\sum_{J,Q}\;\sum_{M=-J}^{M=J}P\left(M,Q,J\right)\;\nu ,$$

quantifies the average number of unpaired particles at temperature *T*. For full superconductivity, we have 1−X=0.

In our construction, excited multiplets (Q>0) embody paired fermions; *X* measures the population-weighted pair content and thus cleanly distinguishes “few overlapping pairs” (BCS-like) from “many tight pairs” that are BEC-like within our finite system and without spatial coherence-length data. The Gibbs weights, P(J,Q,M), make those multiplets thermally accessible, letting *T* play the role of the tuning knob.

Physically, *X* represents the normalized population of the paired subspace and thus provides a direct observable analogue of the superfluid fraction, quantifying how many fermions participate in collective pairing rather than being free or uncorrelated.

### 2.7. Susceptibility

In physics, susceptibility quantifies a material’s response to an applied field. It is a property that describes how readily a material becomes polarized in an electric field (electric susceptibility) or magnetized in a magnetic field (magnetic susceptibility). High susceptibility indicates a strong response, while low or zero susceptibility suggests a weak or nonexistent response. A useful pairing susceptibility is the temperature slope:

(30) $$\chi \left(T\right)=\frac{dX}{dT},$$

for which its absolute maximum marks the temperature of the fastest change in the pair content.

Primary Marker: χ(T) identifies the temperature $T_{X}^{★}$ at which |χ(T)| attains its maximum. This corresponds to the point of fastest growth in X(T) and indicates the rapid reorganization of the microstate population from low-*Q* (BCS-like) to high-*Q* BEC-like sectors.

Physically, *X* represents the normalized population of the paired subspace and thus provides a direct observable analogue of the superfluid fraction, quantifying how many fermions participate in collective pairings rather than being free or uncorrelated. Among possible response functions, χ(T) was chosen because it provides the clearest signal of pair restructuring. Other derivatives such as dS/dT and $dU_{4}/dT$ peak at nearby temperatures (ΔT/T<0.05), confirming the consistency of crossover markers.

### 2.8. Binder Cumulants

We remind the reader that in probability theory, a cumulant is a statistical measure that describes the characteristics of a probability distribution, similarly to moments. They are particularly useful because they are simplified when random variables are added, as the cumulants of the sum of independent variables are simply the sums of individual cumulants. The first few cumulants correspond to the mean, variance, skewness, and kurtosis of a distribution.

The Binder cumulant (often denoted $U_{4}$) is a dimensionless quantity introduced by Kurt Binder to study phase transitions and finite-size scaling in statistical physics. It is essentially a normalized fourth-order cumulant of an order parameter (e.g., magnetization, density of pairs, etc.) [33]. For an order parameter θ, the Binder cumulant is defined as follows:

(31) $$U_{4}=1-\frac{\langle \theta^{4}\rangle }{3{\langle \theta^{2}\rangle }^{2}}.$$

#### Why Is It Useful?

Let us answer this question with a list, which is as follows:

1. Detecting Phase Transitions: Near a critical point, the probability distribution of the order parameter changes shape (from broad/Gaussian-like to peaked/non-Gaussian). $U_{4}$ captures this change: (a) in a disordered (Gaussian) regime, $U_{4}\approx 0$; (b) in an ordered regime, $U_{4}>0$; (c) at criticality, different system-size $U_{4}$ curves cross at the critical temperature, producing a size-independent signature.
2. Finite-Size Scaling: Because it is dimensionless, $U_{4}$ has weak dependence on the system’s size, making it ideal for locating critical points in finite systems.
3. The numerator $\langle \theta^{4}\rangle$ measures the “fatness” of the tails of the distribution of the order parameter. The denominator normalizes it by the variance squared. If the distribution is Gaussian, the ratio is exactly 3, giving $U_{4}=0$. Deviations from Gaussianity—which occur near phase transitions—show up as nonzero $U_{4}$. Thus, in our context, by applying the Binder cumulant to the number of paired fermions, we are probing whether the distribution of pair numbers is broad and Gaussian (weak correlations) or strongly peaked/non-Gaussian (strong correlations and crossover physics).

Secondary Marker: To capture changes in the shape of the distribution, we define the Binder-like cumulant [33]

(32) $$U_{4}\left(T\right)=1-\frac{\langle {\left(Q-\langle Q\rangle \right)}^{4}\rangle }{3\;{\langle {\left(Q-\langle Q\rangle \right)}^{2}\rangle }^{2}},$$

which tends to 2/3 for sharply peaked distributions and to 0 for broad, nearly Gaussian ones.

### 2.9. Statistical Complexity LMC

Given the Gibbs canonical probability distribution (26), the López-Ruiz–Mancini–Calbet (LMC) statistical complexity is defined as the product of two distinct contributions [34]:

(33) C=S·D,

where *S* denotes Boltzmann entropy, which quantifies the information content (or uncertainty) of the following distribution:

(34) $$S=-k_{B}\sum_{J,Q}\sum_{M=-J}^{J}P\left(M,Q,J\right)\log P\left(M,Q,J\right),$$

with the Boltzmann constant set to $k_{B}=1$. The second term, *D*, represents disequilibrium, which measures the departure of the distribution from the uniform case and captures its structural organization:

(35) $$D=\sum_{J,Q}\sum_{M=-J}^{J}{\left(P\left(M,Q,J\right)-\frac{1}{2(2j+1)}\right)}^{2}.$$

The disequilibrium *D* is defined following the LMC measure as $D=\sum_{i}{\left(p_{i}-1/\Omega \right)}^{2}$, quantifying the deviation of the occupation probability $p_{i}$ from uniformity. This metric vanishes for complete equiprobability and increases as the system approaches structural order, serving as a complement to entropy [34].

## 3. Detection Protocols

In SU(2)×SU(2) formalism, we have defined, in Equation (28), the pairing quantifier. Using N=2Ω, this becomes

(36) $$X=\frac{2\langle Q\rangle }{N}=\frac{\langle Q\rangle }{\Omega },$$

such that X∈[0,1] is the fraction of maximally possible pairs in the thermal state.

The pairing susceptibility is defined in Equation (30), for which its largest magnitude identifies the temperature of the fastest change in X(T).

Recall that the Binder-like cumulant of the pair content is defined in Equation (31), which is sensitive to the shape of the distribution.

### Crossover Detection Protocol

The BCS→BEC-like crossover can be diagnosed via the following:

1. Locate $T_{X}^{★}$, where |χ(T)| is maximal; this signals the rapid reorganization from low-*Q* to high-*Q* sectors.
2. Check $U_{4}\left(T\right)$ for different *N* values; intersections give an *N*-insensitive crossover estimate.
3. Classify regimes using thresholds $X_{\mathrm{BCS}}$ and $X_{\mathrm{BEC}}$ (e.g., 0.2 and 0.8).

All quantities are directly computable from P(J,Q,M) without requiring spatial coherence-length information.

## 4. Results

**Figure 1:**
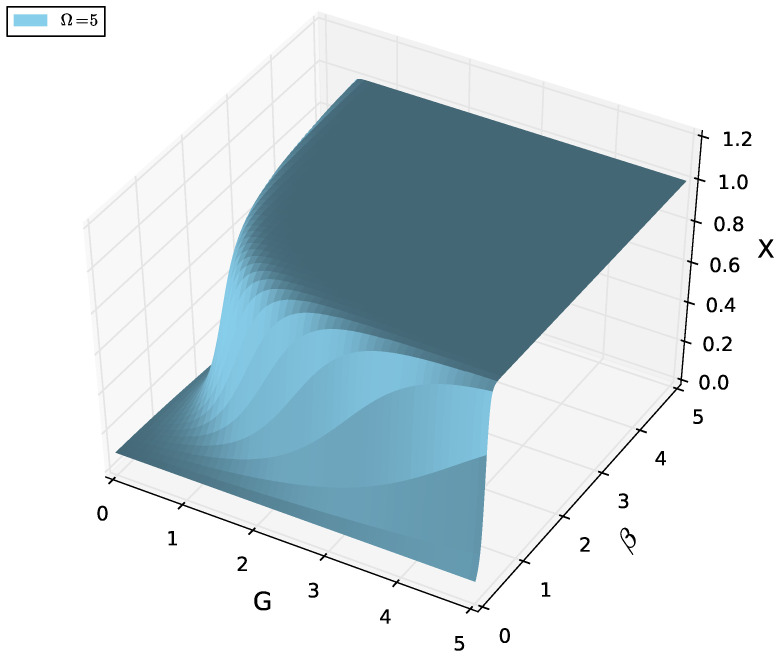
Effective superconductivity index *X* as a function of the pairing strength *G* and the inverse temperature β for Ω=5. *X* measures the fraction of paired fermions, interpolating smoothly between the unpaired regime (X≈0) and the fully paired regime (X≈1). At weak coupling *G*, *X* remains small even at low temperatures, while at strong coupling and large β, the system saturates near X=1, corresponding to a BEC-like paired state. The steepest gradients of the surface mark the finite-size analogue of the BCS-BEC crossover, where thermal fluctuations and pairing correlations compete to reorganize the system from weakly bound to strongly bound pairs.

Figure 1 below nicely illustrates the present scenario: It represents the thermal suppression of pair coherence, serving as a geometric indicator of the crossover. Recall that *X* given in Equation (36) measures the fraction of paired fermions (X=0: no pairs; X=1: fully paired). It is our “effective superconductivity index”. Points to be made in this respect follow below.

1. Dependence on *G* (pairing strength): For small *G*, *X* remains close to zero at all temperatures, since the system cannot sustain pairings without strong coupling. As *G* increases, *X* grows monotonically, approaching saturation (X→1) for large *G*, signaling that most particles are bound into pairs. This is the standard BCS-BEC trend: stronger attraction favors tightly bound pairs.
2. Dependence on β: For low β (high *T*), *X* is suppressed across all couplings, because thermal agitation breaks pairs. As β grows (temperature decreases), *X* increases significantly, especially at intermediate–large *G*. At high β, the surface flattens near X≈1 for strong couplings, indicating a nearly pure paired ground state.
3. Crossover Behavior: The surface X(G,β) shows a crossover ridge; at intermediate *G*, the slope of *X* with respect to β is steepest. This corresponds to the finite-size analogue of a BCS-BEC transition line: Weakly bound pairs emerge gradually as *G* is increased, and thermal suppression competes with pairing. This “ridge” identifies the region where the system reorganizes structurally, shifting from mostly unpaired to mostly paired fermions.
4. Finite-Size Effects (Ω=5): Because the system is small, *X* never exhibits a sharp jump but rather a smooth crossover surface. Interestingly, even for moderate *G*, increasing β (cooling) can drive *X* toward large values, showing that temperature alone can effectively induce BEC-like behavior [32].

Summing up, the figure demonstrates that *X* interpolates smoothly between the unpaired regime (X≈0) and the fully paired regime (X≈1), with the transition controlled both by coupling *G* and inverse temperature β. Strong coupling and low temperatures reinforce each other in stabilizing pairs, while weak coupling or high temperatures suppress pairing. The steepest slopes of the surface mark the finite-size analogue of the BCS-BEC crossover region.

For the following figures, we set N=16. The results for N=8−24 exhibit indistinguishable normalized trends; the choice N=16 offers a convenient balance between computational simplicity and spectral richness, ensuring graphical clarity.

**Figure 2:**
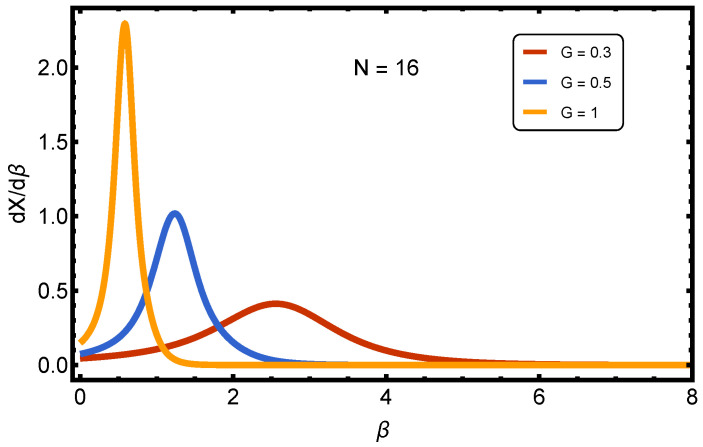
(Color online) Derivative of the superconductivity index, dX/dβ, as a function of inverse temperature β for N=16 and three values of the pairing strength *G*. The peaks signal the temperature $T_{X}^{★}$ where pairing correlations change most rapidly, marking the finite-size analogue of the BCS-BEC crossover. For weak coupling (G=0.3), the response is negligible; for intermediate coupling (G=0.5), a broad peak indicates a smooth crossover near β∼1, while for strong coupling (G=1), a sharp peak at a lower β shows that pairing is stabilized already at relatively high temperatures.

Figure 2 refers to the derivative dX/dβ versus β and three values of the pairing strength *G*. The points to be made follow below:

1. General Meaning of dX/dβ: Since *X* measures the degree of pairing (normalized between 0 and 1), its derivative with respect to β highlights the temperature sensitivity of the pairing. The peaks in dX/dβ indicate the inverse temperature $\beta^{★}$ (or, equivalently, temperature $T^{★}$) at which the system undergoes the most rapid restructuring of pairing correlations, i.e., the crossover region between BCS-like weak pairings and BEC-like strong pairings.
2. Observed Behavior by Coupling *G*: (a) Weak Coupling (G=0.3, red curve): The response is very low; dX/dβ remains close to zero. This means that in the weak-pairing regime, changes in temperature hardly affect the pairing fraction—the system remains essentially unpaired. (b) Intermediate Coupling (G=0.5, blue curve): A clear peak develops near β∼1. This indicates that, at this coupling strength, the system exhibits a pronounced crossover: As temperature is lowered, *X* rises quickly, and the system reorganizes toward stronger pairings. (c) Strong Coupling (G=1, orange curve): The peak is much sharper and located at lower β (higher temperature). This shows that when the pairing is strong, even moderate cooling is enough to lock most fermions into pairs, and the transition to a BEC-like regime happens earlier.
3. Physical Interpretation: The location of the maxima in dX/dβ defines the crossover line $T_{X}^{★}\left(G\right)$ in the (β,G) plane. The height of the peaks indicates the sharpness of the transition: stronger coupling produces a steeper, more abrupt rise of *X*. Thus, the figure demonstrates that the BCS-BEC crossover is temperature-driven at fixed *G* and that its position shifts systematically with the strength of pairing interaction.

In short, weak coupling shows negligible temperature-driven changes in pairings, intermediate coupling exhibits a smooth crossover around β∼1, and strong coupling leads to a sharp, high-temperature crossover. The peaks of dX/dβ serve as finite-size markers of the BCS-BEC crossover temperature.

4. Dependence on *G* (pairing strength): For low *G*, *X* remains close to zero at all temperatures, since the system cannot sustain pairing without a strong coupling. As *G* increases, *X* grows monotonically, approaching saturation (X→1) for large *G*, signaling that most particles are bound into pairs. This is the standard BCS-BEC trend: stronger attraction favors tightly bound pairs.
5. Dependence on β: For low β (high *T*), *X* is suppressed across all couplings, because thermal agitation breaks pairs. As β grows (temperature decreases), *X* increases significantly, especially at intermediate–large *G*. At high β, the surface flattens near X≈1 for strong couplings, indicating a nearly pure paired ground state.
6. Crossover Behavior: The surface X(G,β) shows a crossover ridge: At intermediate *G*, the slope of *X* with respect to β is the steepest. This corresponds to the finite-size analogue of a BCS-BEC transition line: Weakly bound pairs emerge gradually as *G* is increased, and thermal suppression competes with pairing. This “ridge” identifies the region where the system reorganizes structurally, shifting from mostly unpaired to mostly paired fermions.
7. Finite-Size Effects: Because the system is small, *X* never exhibits a sharp jump but rather a smooth crossover surface. Interestingly, even for moderate *G*, increasing β (cooling) can drive *X* toward large values, showing that temperature alone can effectively induce BEC-like behavior [32].

**Figure 3:**
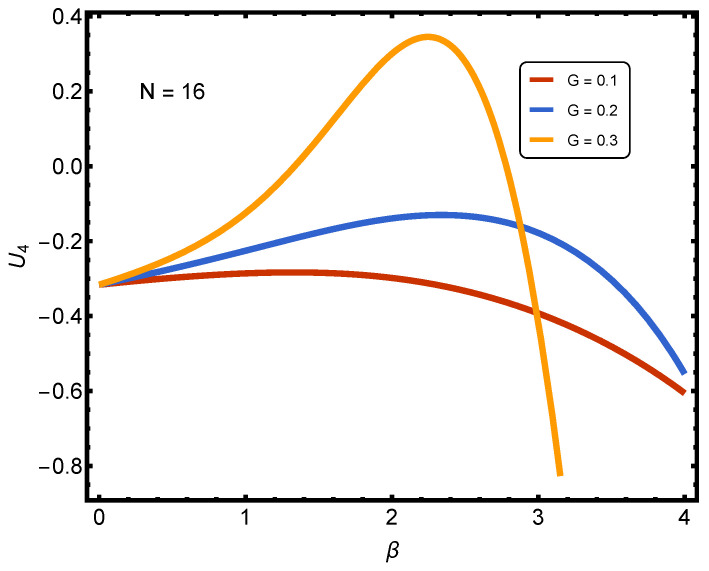
(Color online) Binder cumulant $U_{4}$ of the number of paired fermions for N=16 as a function of inverse temperature β and different interaction strengths *G*. The first figure (weaker couplings, G=0.1,0.2,0.3) shows that $U_{4}$ remains negative or only weakly rises towards zero, indicating nearly Gaussian fluctuations of pair numbers with only modest signs of correlation.

**Figure 4:**
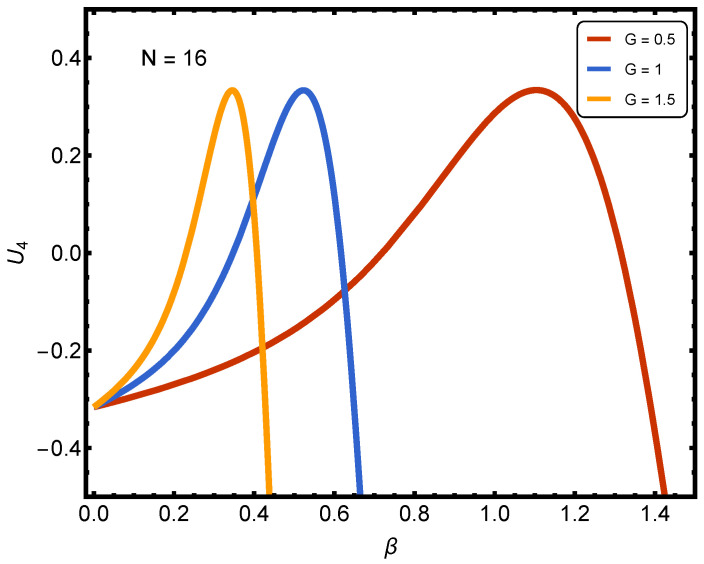
(Color online.) In contrast to what is depicted in Figure 3, Figure 4 (intermediate-to-strong coupling, G=0.5,1.0,1.5) exhibits a clear qualitative change: $U_{4}$ develops pronounced positive peaks at progressively smaller β as *G* increases. This progression demonstrates that stronger pairing correlations drive the emergence of non-Gaussian, ordered fluctuations at higher temperatures, providing a finite-size diagnostic of the BCS-BEC crossover.

Figure 3 and Figure 4 refer to Binder cumulants for our problem. The *y* axis displays the Binder cumulant $U_{4}$ of the number of paired fermions, while the *x*-axis represents the inverse temperature β. The curves correspond to different pairing interaction strengths.

In Figure 3, we note the following features according to different colors: (1) red: G=0.1 (weak); (2) blue: G=0.2 (less weak); (3) orange: G=0.3 (intermediate). In contrast, the horizontal dashed line marks $U_{4}=0$. Our interpretation is as follows:

1. Recall that the Binder cumulant $U_{4}$ captures the shape of the probability distribution of the order parameter (here, the number of paired fermions). A negative $U_{4}$ entails broad/nearly Gaussian-like fluctuations (no sharp ordering). A positive $U_{4}$ entails more peaked, non-Gaussian fluctuations (signatures of emerging collective order or crossover).
2. At weak coupling G=0.1, $U_{4}$ remains negative and relatively flat across all β values. This suggests no significant structural change in fluctuations of pair numbers: the distribution remains broad and Gaussian-like.
3. At less weak coupling G=0.2, $U_{4}$ rises with β, approaching less negative values, but it stays below zero. This indicates some narrowing of the fluctuations with increasing correlation, but this is not enough to produce strongly ordered distributions.
4. At intermediate coupling G=0.3, $U_{4}$ shows a pronounced rise, crossing into positive values near β∼2 before dropping steeply at higher β. The positive peak signals the onset of strongly correlated pairings, where the distribution of pair numbers departs significantly from Gaussian values and becomes more sharply structured. The subsequent drop may reflect over-constraining at very low temperatures (high β), where fluctuations collapse due to the finite system size.

Key Message of Figure 3: The plot shows that for very weak pairings, the Binder cumulant remains negative, reflecting nearly Gaussian fluctuations. As coupling increases, $U_{4}$ rises and even becomes positive, revealing the emergence of non-Gaussian, ordered fluctuations in the number of fermion pairs. The crossing of $U_{4}$ through zero and the presence of a positive peak provide a diagnostic signal of the BCS-to-BEC crossover regime in this finite-*N* system.

Let us now focus on Figure 4. The curves are as follows: (1) red: G=0.5; (2) blue: G=1.0; (3) orange: G=1.5; dashed line at $U_{4}=0$. We face, again, a general trend compared to the weaker *G*s in Figure 3. For weak couplings, the cumulant stayed mostly negative or had modest peaks. Here, with stronger couplings, all curves rise sharply with β, cross into positive values, and show distinct peaks before dropping again. This signals that the system develops more pronounced non-Gaussian fluctuations in the number of pairs. Our interpretation is as follows:

1. At G=0.5, we observe a smooth rise, a peak near β≈1.0, and then a decay. This indicates the onset of collective behavior at lower temperatures rather than weak coupling, but the onset is still broad compared to larger *G*.
2. At G=1.0, we see a much sharper peak at lower β≈0.5. This suggests that stronger pairing drives the earlier emergence of ordered fluctuations, consistent with a crossover towards a BEC-like paired regime.
3. At G=1.5, the peak is even sharper and occurs at an even lower β≈0.3. This shows that the stronger the coupling, the earlier (at higher temperature) the cumulant develops strong positive values, signaling robust pair correlations. The steep fall after the peak reflects the collapse of fluctuations in the small finite system size.

The key physical message of our plots is the following: Increasing *G* shifts the Binder cumulant peaks to smaller β (higher temperature), showing that pairing correlations set in earlier as the interaction strength grows. The positive peaks of $U_{4}$ highlight strong, non-Gaussian fluctuations characteristic of collective pairing behaviors. The progressively sharper and earlier peaks with growing *G* are a clear finite-size signature of the BCS-BEC crossover physics: from smooth Gaussian-like fluctuations at weak coupling to sharply ordered, non-Gaussian ones at strong coupling.

The Binder cumulant curves for different system sizes display near-crossing behavior without a unique intersection, consistent with a smooth, finite-size crossover rather than a sharp phase transition.

**Figure 5:**
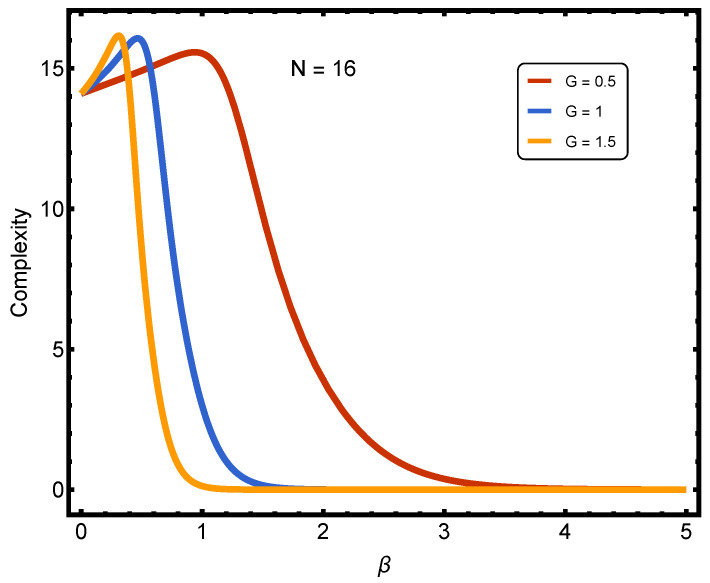
(Color online) Statistical complexity *C* as a function of the inverse temperature β for N=16 and three values of the pairing strength *G*. The results show that the complexity evolves with *G* roughly as $U_{4}$ in Figure 4. This is a new result in information theory.

In Figure 5, we present the behavior of statistical complexity *C* for the number of paired fermions. The objective of this figure is to show that $U_{4}$ behaves similarly to LMC statistical complexity, representing a novel finding. The results indicate that *C* evolves with pairing strength *G* in a manner similar to $U_{4}$.

For a representative system size of N=16, the temperature dependence of *X*, χ, and $U_{4}$ reveals a smooth BCS-BEC crossover. Calculations for N=8 to 24 confirm that normalized observables are nearly size-independent; N=16 is displayed for clarity. The coupling constant *G* shown in Figure 2, Figure 3, Figure 4 and Figure 5 corresponds to weak, intermediate, and strong regimes chosen to illustrate gradual, intermediate, and saturated pair correlations, respectively.

The Binder cumulant curves for different *N* values exhibit near-crossing behavior but do not intersect at a single point, confirming the absence of a true critical temperature and establishing the smooth, finite-size nature of the crossover. Figure 5 consolidates the three diagnostics, *X*, χ, and $U_{4}$, demonstrating their correlated evolution on a common temperature scale.

## 5. Discussion

The stabilizing effect of low temperatures and strong couplings observed here parallels the noise-enhanced stability effect known in stochastic dynamics, where fluctuations extend the lifetime of metastable states. Both phenomena share the feature of fluctuation-driven trapping, though arising from different mechanisms. The analogy underscores how thermal noise can assist rather than hinder order formation.

Our results demonstrate that the temperature-induced restructuring of fermionic correlations mirrors classical fluid crossovers, bridging quantum pairings with thermodynamic criticality concepts. By connecting these findings with the well-established BCS-BEC crossover literature, the present work introduces temperature as an effective tuning parameter, even in finite canonical systems, thus broadening the conceptual map of crossover physics.

In summary, our results show the following:

- The temperature at which the susceptibility χ reaches its maximum scale is in agreement with the known finite-size reduction in critical temperatures [24,35,36,37].
- The entropy and Binder cumulant reproduce the characteristic entropy-flattening phenomenon and non-Gaussian fluctuations seen across the crossover.
- The change in the sign of the effective chemical-potential proxy, inferred from the curvature of X(T), mirrors the transition μ→0 that marks the onset of pair condensation.

Finally, we emphasize that our goal is to demonstrate that temperature alone can drive a structural crossover analogous to the BCS-BEC transition even at fixed coupling.

## 6. Conclusions

In this work, we have explored the behavior of the quantifier *X*, its susceptibility χ, and the Binder cumulant $U_{4}$ in an exactly solvable SU(2) fermionic pairing model, with the aim of diagnosing the crossover from the BCS to BEC regimes. Our results show that these statistical indicators provide complementary signatures of the transition. While *X* captures the progressive restructuring of fermionic correlations, its susceptibility χ reveals the loci of enhanced fluctuations, and the Binder cumulant $U_{4}$ highlights the emergence of non-Gaussian features in the distribution of paired particles.

Taken together, these diagnostics illustrate how finite-size systems encode precursor signs of the BCS-BEC crossover, even before the thermodynamic limit is approached. This highlights the utility of finite-size scaling tools and information-theoretic quantifiers in understanding pairing phenomena across regimes. Moreover, the unified treatment developed here can be readily adapted to other many-body contexts where crossovers and structural transitions play a central role.

Beyond its specific application to the pairing Hamiltonian, our analysis underscores the broader message that information-geometric and fluctuation-based methods provide sensitive, model-independent probes of structural changes in quantum many-body systems. We expect that the present approach will stimulate further work on bridging traditional condensed matter diagnostics with modern information-theoretic and statistical tools.

In short, our findings demonstrate that information-theoretic diagnostics can act as early and precise markers of crossover physics, offering a powerful complement to conventional condensed-matter approaches.

This study situates finite-size quantum pairings within the broader landscape of fluctuation-enabled order, resonating with recent developments in noise-assisted quantum control and complex-system thermodynamics.

## Data Availability

No new data were created or analyzed in this study.
